# Concentration-Dependent Activity of Pazufloxacin against *Pseudomonas aeruginosa*: An In Vivo Pharmacokinetic/Pharmacodynamic Study

**DOI:** 10.3390/antibiotics11070982

**Published:** 2022-07-21

**Authors:** Yasuhiro Umezaki, Kazuaki Matsumoto, Kazuro Ikawa, Yuta Yokoyama, Yuki Enoki, Akari Shigemi, Erika Watanabe, Koyo Nakamura, Keiichiro Ueno, Hideyuki Terazono, Norifumi Morikawa, Yasuo Takeda

**Affiliations:** 1 Department of Clinical Pharmacy and Pharmacology, Graduate School of Medical and Dental Sciences, Kagoshima University, Kagoshima 890-8520, Japan; yume@fukuoka-u.ac.jp (Y.U.); matsumoto-kz@pha.keio.ac.jp (K.M.); yokoyama-yt@pha.keio.ac.jp (Y.Y.); enoki-yk@pha.keio.ac.jp (Y.E.); a-shige@m3.kufm.kagoshima-u.ac.jp (A.S.); erikh2020@yahoo.co.jp (E.W.); nakamura01090@gmail.com (K.N.); k-6ueno@m3.kufm.kagoshima-u.ac.jp (K.U.); terazono@m.kufm.kagoshima-u.ac.jp (H.T.); 2 Department of Clinical Pharmacotherapy, Hiroshima University, Hiroshima 734-8551, Japan; ikawak@hiroshima-u.ac.jp (K.I.); morikawa@hiroshima-u.ac.jp (N.M.)

**Keywords:** pharmacokinetics, pharmacodynamics, pazufloxacin, *Pseudomonas aeruginosa*, murine thigh infection model

## Abstract

The bacterium *Pseudomonas aeruginosa* is known to be associated with nosocomial infections around the world. Pazufloxacin, a potent DNA gyrase inhibitor, is known to be an effective drug candidate. However, it has not been clarified whether the pharmacokinetic (PK)/pharmacodynamic (PD) of pazufloxacin was effective against *P*. *aeruginosa*. Herein, we demonstrated that the PK/PD index of pazufloxacin against *P*. *aeruginosa* infection is used to optimize the dosing regiments. We constructed an in vivo infection model by infecting *P*. *aeruginosa* into the thigh of a mouse to determine the PD, and we measured the serum concentration of pazufloxacin to construct the PK model using high-performance liquid chromatography. The therapeutic efficacy of pazufloxacin was correlated with the ratio of the area under the free concentration time curve at 24 h to the minimum inhibitory concentration (*f*AUC_24_/MIC), and the maximum free concentration to the MIC (*f*C_max_/MIC). Each contribution rate (R^2^) was 0.72 and 0.65, respectively, whereas the time at which the free drug concentration remained above the MIC (R^2^ = 0.28). The target value of pazufloxacin *f*AUC_24_/MIC for stasis was 46.1, for 1 log_10_ it was 63.8, and for 2 log_10_ it was 100.8. Moreover, *f*C_max_/MIC for stasis was 5.5, for 1 log_10_ it was 7.1, and for 2 log_10_ it was 10.8. We demonstrated that the in vivo concentration-dependent activity of pazufloxacin was effective against the *P*. *aeruginosa* infection, and successfully made the PK/PD model sufficiently bactericidal. The PK/PD model will be beneficial in preventing the spread of nosocomial infections.

## 1. Introduction

*Pseudomonas aeruginosa* (*P*. *aeruginosa*) is known to be related to hospital-acquired infections such as urinary tract, pneumonia, surgical site, and bloodstream infections [1,2]. The first-line antibiotic therapy typically used against *P*. *aeruginosa* is carbapenems, but the frequency of carbapenem-resistant strains has been steadily increasing, leading to their increased use [3,4] and the need for alternative treatments. Pazufloxacin is a well-known DNA gyrase inhibitor which demonstrates potent activity against *P. aeruginosa* strains in vitro and effectiveness against the *P. aeruginosa* infection [5,6]. As pharmacokinetic/pharmacodynamic (PK/PD) analyses have demonstrated effectiveness for optimizing dosage regimens, and thereby improving outcomes [7], they have received increasing attention. There are a few papers that calculated the PK/PD parameters for fluoroquinolones using a neutropenic mouse thigh infection model. Liu et al. reported that the target value of enrofloxacin AUC_24_/MIC for stasis was 7.8, for 1 log_10_ it was 10.5, and for 2 log_10_ it was 15.1 [8]. Andes et al. reported that the target value of gatifloxacin AUC24/MIC for stasis was 41.2, for 1 log_10_ it was 72.2, and for 2 log_10_ it was 126 [9]. Zhou et al. reported that the target value of antofloxacin fAUC_24_/MIC for stasis was 38.7, for 1 log_10_ it was 66.1, and for 2 log_10_ it was 147 [10]. The target value may vary among fluoroquinolones. Furthermore, we have shown that the target time above the MIC value differs among cephems [11,12]. Tebipenem has been shown to correlate with AUC/MIC and C_max_/MIC better than the time above MIC, despite the use of carbapenems [13]. Thus, we believe that the target value should be indicated for each drug.

However, a PK/PD analysis of pazufloxacin against the *P*. *aeruginosa* infection has not been conducted, even though it is essential for the optimization regarding dosing regimens. Herein, we evaluated the effectiveness of pazufloxacin in vivo and demonstrated the PK/PD index against *P*. *aeruginosa*.

## 2. Results

### 2.1. Susceptibility Testing of Pazufloxacin

The MIC of pazufloxacin is based on our examination against *P*. *aeruginosa,* where ATCC 27853 was 0.5 µg/mL.

### 2.2. PK of Pazufloxacin

**Figure 1:**
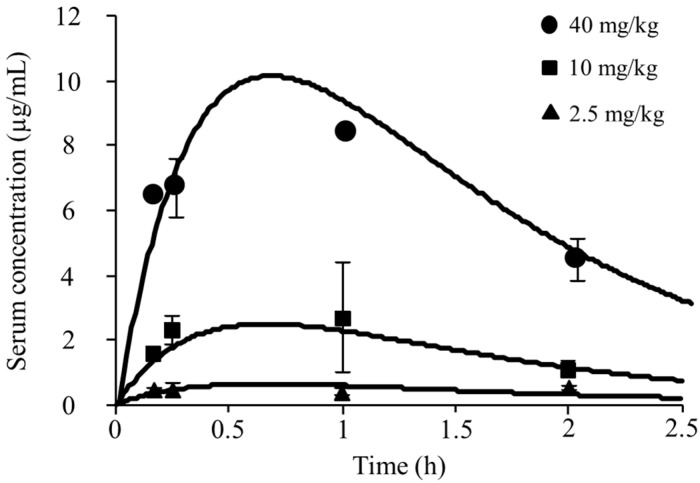
Pharmacokinetic parameters of pazufloxacin after single subcutaneous administration in infected neutropenic mice. Simulation time-concentration curves were created following parameters. Vd = 0.84 L/kg, ke = 2.40 h^–1^, and ka = 0.96 h^–1^. Data are expressed as mean ± S.D. (n = 3).

Figure 1 shows the serum concentrations in the infected neutropenic mice that were administrated pazufloxacin of 2.5, 10, and 40 mg/kg, while Table 1 summarizes the serum PK parameters. The C_max_ ranged from 0.63 to 10.03 µg/mL and the AUC_0__–∞_ from 1.35 to 21.6 µg∙h/mL. The C_max_ and AUC_0__–∞_ showed a linear relationship with the dose of pazufloxacin (data not shown). The mean PK values for these three doses were 0.96 ± 0.25 h^–1^, 2.40 ± 0.55 h^–1^, and 0.84 ± 0.12 L/kg, respectively, and the %PB of pazufloxacin was 20.25 ± 3.88%.

### 2.3. Association between Antibacterial Effects and PK/PD Indices

**Figure 2:**
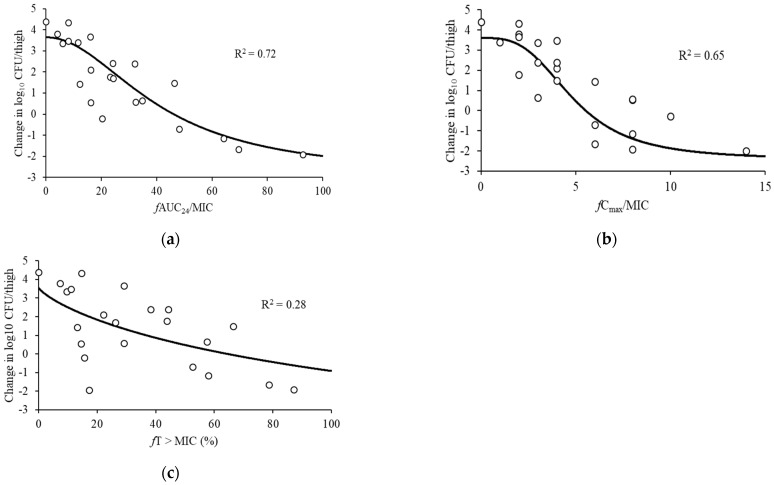
Relationships between the change in log_10_ CFU/thigh at 24 h and PK/PD indices. (**a**) *f*AUC_24_/MIC, (**b**) *f*C_max_/MIC, and (**c**) *f*T > MIC for *P*. *aeruginosa* ATCC 27853. The horizontal dashed lines: organism burden at the start of therapy. Plot: mean for one thigh per mouse. R^2^: coefficient of determination.

The bacterial loads of the thigh-infected animals 2 h after inoculation and the untreated control mice were 4.43 ± 0.18 log_10_ and 8.82 ± 0.13 log_10_ CFU/thigh, respectively. The highest examined doses reduced the bacterial burden to 2.50 ± 0.53 log_10_ CFU/thigh. Figure 2 shows the associations between each of the PK/PD indices and the antibacterial effects for *P*. *aeruginosa* ATCC 27853. In regards to the PK/PD indices for pazufloxacin, stronger correlations were observed between the therapeutic efficacy of pazufloxacin and *f*AUC_24_/MIC (R^2^ = 0.72) and *f*C_max_/MIC (R^2^ = 0.65) compared with *f*T > MIC (R^2^ = 0.28). Table 2 shows the PK/PD model parameter estimated for the *f*AUC_24_/MIC and *f*C_max_/MIC indices for pazufloxacin against *P*. *aeruginosa*. 

### 2.4. Target PK/PD Index in Relation to Efficacy

Based on these results, we summarized the target values of *f*AUC_24_/MIC and *f*C_max_ /MIC required for stasis, 1 log_10_ and 2 log_10_ reductions in the bacterial burden in Table 3. As a result, the target values of *f*AUC_24_/MIC and *f*C_max_ /MIC for a 2 log_10_ kill were required to be two times higher compared to the static effect.

## 3. Discussion

The purpose of this study was to reveal the PK/PD parameters of pazufloxacin, which has exhibited direct antimicrobial activity against *P*. *aeruginosa* as a potent inhibitor of DNA gyrase and bactericidal activity against *P*. *aeruginosa* in in vitro experiments [14].

To predict the clinical efficacy in the PK/PD analyses, the use of a murine infection model, which was established by Craig et al. [15], has become a standard method that is frequently used to optimize doses of antimicrobial agents in clinical trials. The results showed that in neutropenic mice, nonlinearity was a feature of the unbound PK of pazufloxacin. This PK nonlinearity was seen in various dose ranges of pazufloxacin required to fully characterize the PK/PD relationship. Although the number of samples and sampling points used in the PK analysis were 3 and 4, respectively, the PK parameters (Vd = 0.84 L/kg, ke = 2.40 h^–1^ and CL = 2.0 L/h) were consistent with the report by Fukuda et al. [16]. Thus, the PK parameters obtained in this study were considered reasonable. To develop free plasma concentrations for multiple dosing patterns throughout the 24 h treatment period, we applied the superposition principle to the single-dose unbound plasma pazufloxacin concentration time curves. As a result, based on the R^2^ values and a visual examination of the fit, the *f*AUC_24_/MIC and *f*C_max_/MIC ratios in the thigh infection models were more suitable to predict the in vivo bacterial killing than the *f*T > MIC (Figure 2). Previously, the time course of bacterial activity was investigated using the time–kill curve experiment [17]. In the present study, two major bactericidal activity patterns of time- and concentration-dependent killing were observed in the drug concentrations. As concentration-dependent killing has been observed over various concentrations with aminoglycosides and fluoroquinolones, the bactericidal activity pattern of pazufloxacin, which is referred to as concentration-dependent killing, was similar to that of aminoglycosides and fluoroquinolones in the in vitro experiments, as shown in Figure 2. Similar results were observed between these antimicrobial agents in the model mouse using a pazufloxacin-susceptible *P*. *aeruginosa* isolate when the pazufloxacin dosage exceeded the MIC, similar to that of aminoglycosides; this finding verifies the concentration-dependent efficacy of this antimicrobial agent [16]. The most suitable PK/PD parameter for fluoroquinolones in animal models is the AUC_24_/MIC or C_max_/MIC ratio [8,9,10]. In our study, the *f*AUC_24_/MIC and *f*C_max_/MIC ratio required for a static effect and maximum killing of the *P*. *aeruginosa* thigh infection were estimated to be approximately 46.1 and 5.5 and 100.8 and 10.8, respectively (Table 3). The mean C_max_ or AUC_24_ of a single administration of 1000 mg pazufloxacin reported 18.06 mg/L or 58.6 mg∙h/L, respectively [18]. Nakamura K. et al. reported that the Monte Carlo simulation demonstrated that 1000 mg of pazufloxacin administered every 12 h (2000 mg daily) can achieve >90% probability of the target attainment in a patient with prostatic hypertrophy with MIC = 2 mg/L [19]. They reported that a prostatic penetration of pazufloxacin (prostate tissue/plasma ratio) was good at C_max_ (0.82–0.99) and AUC_0–1.5_ (0.80–0.98). In general, tissue penetration of fluoroquinolone antibiotics (e.g., pazufloxacin) were good, and unbound concentrations of pazufloxacin in the tissue’s interstitial fluids are similar to those in plasma [20]. Therefore, we expected that our demonstrated data will improve clinical outcomes in patients with *P*. *aeruginosa* infections. In this study, we clarified the PK/PD parameters of PZFX against *P. aeruginosa*. However, only one strain was used; therefore, further experiments using other strains or clinical isolates is desirable. In addition, the validation of the PK/PD parameters for other Gram-negative bacteria has not been validated, so further investigation is needed.

## 4. Materials and Methods

### 4.1. Materials

*P*. *aeruginosa* was purchased from ATCC (ATCC 27853; ATCC, Rockford, MD, USA). Sheep blood agar was purchased from Nissui Pharmaceutical Co., Ltd. (Tokyo, Japan). Pazufloxacin mesilate was purchased from Taisyo Toyama Pharmaceutical Co., Ltd. (Tokyo, Japan).

### 4.2. Susceptibility Test of Pazufloxacin

We determined the MIC of *P*. *aeruginosa* for pazufloxacin according to the recommended standardized procedure [21]. *P*. *aeruginosa* was cultivated onto sheep blood agar at 37 °C before each experiment. We serially diluted the P. aeruginosa suspension (1.5 × 10^6^ CFU/mL) tenfold with MHB, and then plated 50 µL of each diluent onto Mueller–Hinton agar plates with or without pazufloxacin (Taisyo Toyama Pharmaceutical Co., Ltd., Tokyo, Japan) in each concentration (0.12, 0.25, 0.5, 0.75, or 1 µg/mL) [22], and the MIC of ATCC 27853 was evaluated after 16–20 h.

### 4.3. In Vivo Study

The animal study was preceded by approval from the Kagoshima University Institutional Animal Care and Use Committee (approval No.: MD12106). The development of the neutropenia and thigh infection model mouse was performed according to the previous report [23,24]. Briefly, we intraperitoneally administrated 150 mg/kg of cyclophosphamide for 4 days into ddY female mice (5 weeks old) following administration of 100 mg/kg for 1 day to induce a “neutropenic condition model” before the experimental infection. Next, before bacterial inoculation, the mice were anesthetized with a mixture of medetomidine (0.3 mg/kg), midazolam (4.0 mg/kg), and butorphanol (5.0 mg/kg) [25]. We injected a bacterial suspension from an early logarithmic phase into a posterior thigh (6 × 10^4^ CFU/mL, 100 µL/thigh). Two hours after the bacterial inoculation, the infection had been reproducibly established. 

### 4.4. Serum Concentration of Pazufloxacin

A total of 2.5, 10, and 40 mg/kg of pazufloxacin was subcutaneously administrated to conduct single-dose serum PK analyses in the neutropenic mice. After that, serum samples were obtained at 10, 15, 30, 60, and 120 min after administration. The serum concentration of pazufloxacin was measured using HPLC with a slightly modified previous method [26]. The validation parameters of the chromatography method are indicated in Supplementary Table S1.

### 4.5. Serum Protein Binding of Pazufloxacin

We determined the protein-binding activity using centrifugal filter units as follows [27]. In the first, the serum samples were incubated for 30 min at 37 °C after samples were centrifuged at 2000× *g* for 10 min at 37 °C. The supernatants were filtrated with a 0.2 µm ultrafilter and the concentration of pazufloxacin wad measured using HPLC. We calculated the protein binding percentage (%PB). The equation was as follows:

%PB = [(Cp − Cpuf)/Cp] × 100

where Cp is the pazufloxacin concentration in serum and Cpuf is the pazufloxacin-free concentration in the ultrafiltrate.

### 4.6. PD Analyses of Pazufloxacin 

Two hours after inoculation with ATCC 27853, pazufloxacin (2.5–35 mg/kg) was injected subcutaneously at intervals of 6, 8, 12, and 24 h in 24 different dosing patterns. We then euthanized the treated mice and removed their thighs 24 h after the start of pazufloxacin administration. Next, we collected thigh samples from the untreated mice and homogenized them in 7 mL of sterile saline to count the number of viable cells at 2 and 26 h after bacterial inoculation. The thigh homogenate was serially diluted tenfold with MHB and spread onto bromothymol blue agar, followed by culturing for 24 h (37 °C). Next, the CFU for each thigh was determined, set at a lower limit of 160 CFU/thigh. The same regimens described above were repeated, except for with 23 different dosing patterns, for the thigh-infected animals. Finally, we euthanized the mice after inoculation.

### 4.7. PK/PD Analyses of Pazufloxacin

The following PK parameters, such as the absorption rate constant (ka (h^–1^)), the elimination rate constant (ke (h^–1^)), the volume of distribution (Vd (L/kg)), and AUC, were calculated based on the drug concentration data, using a standard one-compartment model with first-order absorption and elimination processes. The pazufloxacin concentration in the serum (Cp (µg/mL)) at the time (t (h)) after dosing (D (mg/kg)) were described as follows:

Cp = D × ka × (e^–ke^
^× t^ − e^–ka^
^× t^)/Vd (ka − ke)

We then simulated the serum pazufloxacin concentrations based on the mean pharmacokinetic parameters for the four doses to estimate the following three major PK/PD indices: *f*AUC_24_/MIC, *f*C_max_/MIC, and *f*T > MIC.

Then, data regarding the antibacterial activity were fitted to the following model:

E = E0 − (E_max_ × X_γ_)/(EC_50_^γ^ + X_γ_),

where E, E0, and E_max_ represent the killing effect of the pazufloxacin (log_10_ CFU of *P. aeruginosa* per thigh at 24 h), the baseline effect in the absence of the drug, and the maximum killing effect, respectively, where X represents the PK/PD index, EC_50_ represents the PK/PD index value required for 50% of Emax, and γ represents the Hill coefficient describing the steepness of the sigmoid curve. The MULTI program (originally developed by Yamaoka et al. [28], and currently maintained by the Department of Biopharmaceutics and Drug Metabolism, Kyoto University (Kyoto, Japan)) was used for all PK/PD analyses with nonlinear least-squares regression, and the Mann–Whitney U test was used for all statistical analyses.

## 5. Conclusions

We determined the most predictive PK/PD index of pazufloxacin against *P*. *aeruginosa* and the predictive index required for effectiveness. These results would be useful to estimate the optimal dose of pazufloxacin to fight against *P*. *aeruginosa* infections.

## Figures and Tables

**Table 1:** Pharmacokinetic parameters of pazufloxacin after single subcutaneous doses of 2.5, 10 and 40 mg/kg.

Dosing Regimen (mg/kg)	C_max_ (µg/mL)	AUC_0–__∞_ (µg∙h/mL)
2.5	0.63	1.35
10	2.51	5.40
40	10.03	21.6

C_max_, maximum drug concentration; AUC_0**–**__∞_, area under the drug concentration–time curve from zero to infinity.

**Table 2:** Pharmacokinetic/pharmacodynamic (PK/PD) model parameter estimates predicting viable counts at 24 h for the *f*AUC_24_/MIC and *f*C_max_/MIC index of pazufloxacin against *P*. *aeruginosa* in the thigh infection models.

	E_max_ (log_10_ CFU/Thigh)	E_0_ (log_10_ CFU/Thigh)	EC_50_	γ
*f*AUC_24_/MIC	6.56	3.64	41.4	2.04
*f*C_max_/MIC	6.18	3.60	5.54	2.90

*f*AUC_24_/MIC, the ratio of the area under the free concentration–time curve for a 24 h period to the minimum inhibitory concentration; *f*C_max_/MIC, the ratio of the maximum free concentration to the minimum inhibitory concentration; E_max_, maximum killing effect; E_0_, baseline effect in the absence of the drug; EC_50_, PK/PD index value needed for 50% of E_max_; γ, Hill coefficient.

**Table 3:** Target values of pazufloxacin *f*AUC_24_/MIC and *f*C_max_/MIC for a static effect and 1, and 2 log_10_ kill against *P*. *aeruginosa* in the thigh infection models.

	*f*AUC_24_/MIC	*f*C_max_/MIC
static effect	46.1	5.5
1 log_10_ kill	63.8	7.1
2 log_10_ kill	100.8	10.8

*f*AUC_24_/MIC, the ratio of the area under the free concentration–time curve for a 24 h period to the minimum inhibitory concentration; *f*C_max_/MIC, the ratio of the maximum free concentration to the minimum inhibitory concentration.

## Data Availability

Not applicable.

## Supplementary Materials

The following are available online at https://www.mdpi.com/article/10.3390/antibiotics11070982/s1, Table S1: Accuracy and precision of quantification of PZFX.
