# Supplementary material for: Concentration-Dependent Activity of Pazufloxacin against Pseudomonas aeruginosa: An In Vivo Pharmacokinetic/Pharmacodynamic Study
Source: Antibiotics (Basel). 2022 Jul 21;11(7):982. doi: 10.3390/antibiotics11070982 (PMC9312304; doi:10.3390/antibiotics11070982)
Supplement: Supplementary file 1 [file antibiotics-11-00982-s001.zip › antibiotics-1815471-supplementary.pdf]

Table S1 Accuracy and precision of quantification of PZFX

	PZFX concentration (μg/mL)	0.1	1.0	10
Inter-day	Accuracy (%)	-9.95	-7.29	0.07
	Precision (CV, %)	3.59	4.73	0.04
Intra-day	Accuracy (%)	11.19	12.66	-0.13
	Precision (CV, %)	7.77	8.59	9.91
